# Recent Developments on the Performance of Algal Bioreactors for CO_2_ Removal: Focusing on the Light Intensity and Photoperiods

**DOI:** 10.3390/biotech12010010

**Published:** 2023-01-11

**Authors:** Zarook Shareefdeen, Ali Elkamel, Zaeem Bin Babar

**Affiliations:** 1 Department of Chemical and Biological Engineering, American University of Sharjah, Sharjah P.O. Box 26666, United Arab Emirates; 2 Department of Chemical Engineering, University of Waterloo, Waterloo, ON N2L 3G1, Canada; 3 Department of Chemical Engineering, Khalifa University, Abu Dhabi P.O. Box 127788, United Arab Emirates; 4 Institute of Environmental Sciences and Engineering (IESE), School of Civil and Environmental Engineering (SCEE), National University of Sciences and Technology (NUST), Islamabad 44000, Pakistan

**Keywords:** carbon dioxide, algae, greenhouse gas, light intensity, photoperiods, photobioreactors

## Abstract

This work presents recent developments of algal bioreactors used for CO_2_ removal and the factors affecting the reactor performance. The main focus of the study is on light intensity and photoperiods. The role of algae in CO_2_ removal, types of algal species used in bioreactors and conventional types of bioreactors including tubular bioreactor, vertical airlift reactor, bubble column reactor, flat panel or plate reactor, stirred tank reactor and specific type bioreactors such as hollow fibre membrane and disk photobioreactors etc. are discussed in details with respect to utilization of light. The effects of light intensity, light incident, photoinhibition, light provision arrangements and photoperiod on the performance of algal bioreactors for CO_2_ removal are also discussed. Efficient operation of algal photobioreactors cannot be achieved without the improvement in the utilization of incident light intensity and photoperiods. The readers may find this article has a much broader significance as algae is not only limited to removal or sequestration of CO_2_ but also it is used in a number of commercial applications including in energy (biofuel), nutritional and food sectors.

## 1. Introduction

Rising carbon dioxide CO_2_ emissions and their impact on global warming has raised awareness on carbon mitigation technologies. In the recent years, the concept of carbon sequestration or isolating carbon from the atmosphere has gained a great interest. Carbon sequestration can be achieved via direct and indirect means. Direct sequestration entails human-controlled intervention whereas indirect sequestration mostly relies on natural processes. Direct sequestration can be achieved through geological storage and chemical approach. In geological storage methods, CO_2_ is injected deep into geological or oceanic reservoirs. It has been employed in Enhanced Oil Recovery (EOR) process in which gases including CO_2_ are injected into wells to increase oil production. However, direct sequestration has the potential of re-emergence of CO_2_ into the atmosphere which contribute to climate change. Indirect sequestration relies mostly on the ability of natural forest to capture CO_2_ emission from the atmosphere. It includes afforestation (conversion of bare land into forest) and reforestation (replanting of forest land that has previously been lost). Forest-based sequestration is one of the cheapest conventional methods for CO_2_ removal. CO_2_ absorbed by plants are converted into biomass. As the forest matures, the rate of intake of carbon tends to reach a steady state. When old trees die, they decompose, and the CO_2_ released in the process is offset by the growth of new trees. Thus, harvesting a small portion of forest product without impacting the ability of forest to sequester carbon is possible and could further reduce the cost of plantation. However, the limited availability of land makes this approach less significant. Chemical approach to mitigate excessive CO_2_ release into the atmosphere provides a more permanent solution. CO_2_ from flue gas is captured by absorption using a chemical agent and then separated from the absorbing agent for further processing. However, the economic viability of chemical approach is limited. 

Another biological option for CO_2_ removal other than aforementioned techniques, is utilizing microalgae. Microalgae are a diverse group consisting of approximately 50,000 characterized photosynthetic organisms that are naturally found in a wide variety of ecosystems ranging from aquatic to land environments. They lack plant tissues, and can reproduce sexually and asexually (i.e., vegetatively). They offer a much better option for CO_2_ remediation as compared to trees and plants owing to their superior photoautotrophic efficiency [1]. Microalgae can be cultivated in photobioreactors (PBR) where they can be used for CO_2_ fixation under a variety of conditions. In photobioreactors, photosynthesis takes place in which microalgae convert incident light and CO_2_ into biomass. The microalgae can be grown in photobioreactors in either of the three conditions; heterotrophic, autotrophic or combination of both. In heterotrophic conditions, micro-organisms depend on organic carbons as an energy source which allows some of the organisms to grow in complete darkness. In autotrophic conditions, microorganisms use light as an energy source. 

The objective of this work to review recent developments of algal bioreactors for CO_2_ removal with the focus on the effects of light intensity, light incident, photoinhibition, light provision arrangements and photoperiod on the performance of the bioreactors.

## 2. The Role of Algae in CO_2_ Removal

As compared to indirect CO_2_ sequestration, algae-based bioreactor systems require human-controlled intervention. Unlike forest-based sequestration that is often hindered by the availability of land, algae-based approach utilizes land more efficiently and has the potential to provide a superior and sustainable solution considering environment and economics [2]. Since algae could be grown on unproductive land, it has little competition with agricultural land for growing crops. Compared to other terrestrial plants, microalgae provide an excellent solution to ever increasing carbon emission. Their high growth rate, high biomass yield, and efficient utilization of solar energy enhance CO_2_ consumption rates. Vunjak-Novakovic et al. [3], report that the measured removal efficiency of CO_2_ is about 82.3 ± 12.5% on sunny days and 50.1 ± 6.5% on cloudy days. Other than ecological benefits, microalgae have a commercial potential as a carbon-neutral fuel source. Several microalgae species are reported to contain high level of lipid that can be used as a source of biofuel to reduce fossil fuel consumption with simultaneous mitigation of CO_2_ emission. Unlike energy crops such as *Jatropha* and oil palm that require a lot of land, microalgae could be grown in bioreactors or open pond with minimal land with significantly high yield of biofuel [4].

Cultivation of algae could be done either in open ponds or in closed bioreactors. Open ponds are generally cheaper to construct than enclosed bioreactors. The ponds are usually shallow to allow sufficient penetration of light. Open pond is the simplest algae cultivation method, however, the high surface area of open ponds also means that if CO_2_ is bubbled through open ponds, it will diffuse into the atmosphere quickly. This makes open ponds are less suitable for CO_2_ treatment. In addition, there is a higher risk of contamination by other organisms in open ponds that they must be kept highly alkaline. This limits utilization of open ponds to only few algae species that can tolerate high pH levels. Enclosed bioreactors, in comparison, are designed to overcome the limitations of open ponds. Typically, they have higher surface to volume ratio that enhances growth rate and shorten harvest time. More species of algae could also be grown in enclosed bioreactors [5].

Microalgae species are used in bioreactors because they grow faster and have been more thoroughly studied than macro-algae. Among three distinct classes of microalgae (i.e., autotrophic, heterotrophic, and mixotrophic), mixotrophic microalgae yield higher biomass but with lower light intensity requirement; thus making it more energy efficient than purely autotrophic and/or heterotrophic microorganisms. In terms of cellular scale, algae can be divided into macro and microalgae [6]. Macro-algae species are multicellular and are mostly aquatic such as seaweeds. Based on pigmentation, microalgae can be brown, red, and green, and are commonly known as *Phaeophyta*, *Rhodophyta*, and *Chlorophyta*, respectively. On the other hand, microalgae are unicellular, which can either be eukaryotic or prokaryotic. While most ‘true’ microalgae are eukaryotic which means they have a nucleus inside a cell wall. Prokaryotic organisms such as *cyanobacteria* (Blue-green algae) are considered as microalgae due to their photosynthetic abilities. They can also be classified on basis of pigmentation such as green algae, red algae, brown algae, and blue green algae. *Botryococcus brauni* is a green microalga that can tolerate high level of CO_2_ concentrations. The effects of varying amount of CO_2_ concentrations were studied and it was found that productivity of this algal strain reached the highest at CO_2_ concentration of approximately 20% with a maximum biomass concentration of 2.31 g/L after 25 days [7]. With high CO_2_ tolerance and high lipid content of 30–40%, these algae species could be used in many industries and the biomass generated could be used as a feedstock for biofuel production.

*Chlorella vulgaris* is one of the most common algae used in bioreactor. They can grow in mixotrophic, autotrophic or heterotrophic conditions. When grown under mixotrophic conditions, *Chlorella vulgaris* displays the properties of both autotrophic and heterotrophic regimes. They consume organic carbon to augment their growth rate and biomass productivity in a manner similar to heterotrophic cultivation. Additionally, they recycle inorganic carbon in the presence of light to produce O_2_ [8]. Extensive studies among several algal species in the presence of 9% CO_2_, 2.66 kg/day of SO_2_ and 36.25 mg/nm^3^ revealed that *Chlorella vulgaris* had rapid growth rate with excellent oil content and energy recovery of 11.45% and 29.53%, respectively [9]. *Chlorella vulgaris* had been used in commercial-scale photo-bioreactor facility in Klotze, Germany, which was claimed as the world’s largest closed reactor system in 2003. *Chlorella vulgaris* sp. has high sequestration efficiency and has been commercially cultivated for health supplement as it contains valuable substance such as polyunsaturated fatty acids, antioxidants, and minerals [10]. 

Madhubalaji et al. [11] report cultivation of *Chlorella vulgaris* in an airlift photobioreactor. The influence of various factors such as light intensity, photoperiod, and CO_2_ concentrations was studied on the cultivation of microalgae. A specific focus was made on different fatty acids and vitamin B12. Fatty acid production was enhanced by 2 to 3 times in the presence of augmented light intensity and photoperiod. The *Chlorella vulgaris* production was increased to 171 mg L^−1^ day^−1^ at high levels of CO_2_ equivalent to 15% *v*/*v*. On the other hand, the vitamin B12 production was decreased by 30%. 

In another study [12], a membrane-based photobioreactor was developed using optical fibers. A film of *Scenedesmus obliquus* (i.e. green algae species) was linked to the membrane. This facilitated gas permeation and direct assimilation of CO_2_ gas molecules which were diffused through the membrane. The light energy for production of *Scenedesmus obliquus* under autotropic conditions was supplied by an external LED light source. The production of *Scenedesmus obliquus* was examined under various conditions such as quantity of optic fibers and CO_2_ and nitrate levels. Under the operating conditions of nitrate level (1.18 mM), CO_2_ level (10% *v*/*v*), and number of optic fibers (13), the maximum density of *Scenedesmus obliquus* density was 37.32 g m^−2^. 

In a recent study, Chaiklahan et al. [13], investigated the influence of varying light intensity in the range between 635 and 2300 µmol·m^−2^s^−1^ (i.e., here light intensity is expressed in terms of µmol of photons) on the production of *Arthrospira* (*Spirulina*) *platensis* (i.e., multicellular blue-green algae) in a photobioreactor. The semi-continuous operation of the photobioreactor was used to maintain the optical densities (i.e., absorbance) of 0.4, 0.6, and 0.8. At the light intensity of 2300 µmol·m^−2^s^−1^ and optical density of 0.6, the maximum production of *Arthrospira (Spirulina) platensis* was obtained around 0.62 g L^−1^d^−1^. 

Alhaboubi et al. [14] studied comparative performance between a novel three-phase Belt Conveyor Reactor (BCR) and a conventional Airlift Bubble Column Reactor (ALR). In both reactors, *Chlorella vulgaris* was cultivated. Ambient air with CO_2_ level of 0.038% under various aeration rates between 0.145–0.29 vvm (vvm = gas volume per minute/liquid volume in the reactor) was utilized. At the initial concentration of 0.2 g·L^−1^ *Chlorella vulgaris* and aeration rate of 0.29 vvm, the highest biomass production of 2.120 g·L^−1^ (in BCR) and 1.420 g·L^−1^ (in ALR) was found. Additionally, the corresponding CO_2_ abatement efficiencies were 40% and 25%, respectively. In the case of BCR, owing to high gas holdup which increased the mixing quality, the biomass production was nearly 50% more than in the case of ALR. 

In a recent research work performed by Do et al. [15], a novel flat-panel photobioreactor (FPP) was utilized to augment the biomass and lutein yields and CO_2_ removal efficiency by employing *Chlorella sorokiniana* TH01 (freshwater green microalga). Lutein is a type of organic pigment, carotenoid which is related to beta-carotene and vitamin A. At the optimum CO_2_ levels, light intensity, and aeration rate of 5%, 150 µmol·m^−2^·s^−1^, and 1 L·min^−1^, respectively, the maximum biomass and lutein productions and CO_2_ removal efficacy were 284–469 mg·L^−1^·d^−1^, 2.57–4.57 mg·L^−1^·d^−1^, and 63–100%, respectively. 

In Alarde [16], a 2-compartment with a 6L vertical-column photobioreactor was used for the removal of CO_2_ from air by microalgae. The production of microalgae was monitored using temperature, pH, and CO_2_ sensors. Two 7-day trials runs were performed with an initial algae mass of 15 g. It was found that in trial 1 and 2, the biomass yield was 21.5 g and 19.7 g, respectively. In the case of trial 1, the corresponding CO_2_ sequestration rate was increased from 10.17% to 22.04%. Furthermore, it was observed that the CO_2_ sequestration rate and microalgae production rate were directly proportional to each other. 

In a recent study, Senatore et al. [17] utilized *Chlorella vulgaris* for CO_2_ sequestration in a photobioreactor. The influence of photosynthetic photon flux densities (PPFD) (60 and 120 μmol·m^−2^·s^−1^), liquid/gas ratio (L/G = 5, 10 or 15), and CO_2_ concentration (5, 10 and 15%) on CO_2_ capture and biomass growth was examined. It was found that PPFD substantially increased the biomass growth. The highest biomass concentration of 1.01 g·L^−1^ was noticed at PPFD and L/G of 120 μmol·m^−2^·s^−1^ and 15, respectively with CO_2_ abatement efficacy of 80%. Furthermore, the photobioreactor comprised of a novel self-forming dynamic membrane (SFDM) yielded a biomass harvesting rate of 41 g·m^−2^·h^−1^.

A novel mathematical model was developed by Feng et al. [18] and this model simulates the algal production for an indoor membrane bioreactor (A-MBR) which was based on *Chlorella* sp. cultivation. The model incorporates the influence of light intensity on the growth. In addition, Solids Retention Time (SRT) was also considered in the model. The model was applied to estimate the maximum production rates in A-MBR. In the case of indoor and outdoor A-MBRs, the maximum production rates were found to be 6.7 g·m^−2^ d^−1^ and 28 g·m^−2^·d^−1^, respectively. The corresponding light intensities and SRTs: 5732 lux and ~8 d (indoor) and 28,660 lux and ~4 d (outdoor), respectively. The unit ‘lux’ represents the amount of light (lumens) per square meter.

In Ratomski et al. [19], 100 L photobioreactors were utilized to explore the potential of sodium bicarbonate as a cheap CO_2_ alternate for the production of *Chlorella vulgaris* and lipid using gravimetric and spectrophotometric techniques and a well-known Bligh and Dyer method, respectively. A substantial improvement in biomass production was noticed in the presence of bicarbonate in comparison with the samples in the absence of bicarbonate. The maximum concentration of *Chlorella vulgaris* was 572 ± 4 mg L^−1^ at a production of 7.0 ± 1.0 mg L^−1^ d^−1^ and the corresponding bicarbonate concentration, lipid content, and CO_2_ removal rate were 2.0 g L^−1^, 26 ± 4%, and 0.925 ± 0.073 g L^−1^ d^−1^, respectively. 

Beigbeder et al. [20] cultivated 25 various strains of microalgae in a 3.5 L bubble column reactor. Prior to cultivation, these strains were shortlisted in accordance with their capability to grow in a specific range of CO_2_ levels. The influence of important parameters including light-dark cycles and CO_2_ levels (0.04–10%) on the production of strains’ growth and CO_2_ removal was analyzed. The cultivation of *Parachlorella kessleri* microalgae in the presence of 2.5% (*v*/*v*) of pig manure combined with 5% CO_2_ and light/dark cycle of 20 h/4 h generated the highest specific growth rate of 0.58 d^−1^, biomass productivity of 104 mg L^−1^ d^−1^ and CO_2_ fixation rate of 211 mg CO_2_ L^−1^ d^−1^. The corresponding CO_2_ removal rate was found to be 211 mg CO_2_ L^−1^ d^−1^. 

In a study performed by Cui et al. [21], a 15 L hanging bag (reactor) was used. This is said to be the very first study to analyze the suitability of hanging bag system in the technological domain of photobioreactors. The study employed new design of spargers to characterize the bubbling property. Additionally, other important parameters such gas hold-up and mixing time and CO_2_ mass transfer coefficient were also explored. A remarkable improvement (up-to two times) in the production of *Chlorella Sorokiniana* was noticed at a gas flow rate of 5 L min^−1^ by employing new sparger design. Furthermore, *Chlamydomonas reinhardtii* production in this reactor unit was also increased by 11%. The modifications strongly suggest that the proposed simplistic hanging bag system significantly increased micro-algal productivity in a cost effective manner. 

Like other *Chorella* strains, *Dunaleilla tertiolecta* also has a high growth rate and can be harvested. As growth rate is proportional to CO_2_ consumed, the faster the algal growth, the more efficient the removal of CO_2_ from the flue gas. Carbon removal efficiency of about 80% during sunny day and 50% during cloudy days was achieved in a pilot study using *Dunaleilla* strain with 30% of culture harvested daily [3]. *Porphyridium* sp. is a red microalga that grows in seawater.

Microalgae with an interesting anatomy, *Oscillatoria* sp. cells are known for their continuously ‘oscillating’ filaments. In a study [22], *Oscillatoria* sp. was used for CO_2_ fixation, in an environment of even 100% CO_2_ with a photoperiod of 12:12. A very reasonable CO_2_ fixation of >64% was reported. 

A multi-algae consortium was also used for CO_2_ sequestration. In a study [23], *Ankistrodesmus* sp. was cultivated in a mix culture along with *Chlorella* sp. and *Scenedesmus* sp. with an incident light intensity of 4000 lux with 16:8 hr photoperiod. CO_2_ removal efficiency of up to 59.8% and CO_2_ removal rate of up-to 979.62 mg L^−1^d^−1^ were reported. 

In addition to aid in CO_2_ removal, microalgae offer other value added products. As previously highlighted, they can be converted to commercially viable products such as biofuels, chemicals, and cosmetics. Several microalga species can be used for synthesis of proteins and Provitamin-A which are frequently used in pharmaceuticals. A few microalgae are also capable of nitrogen fixation, which allow their use in paddy growth. In a study [24], it was found that 0.4 gm of new biomass was produced for each 1 gm of CO_2_ fixation when *Euglena gracilis* was used for CO_2_ sequestration in a photobioreactor. The harvested biomass was recommended for animal feed, owing to its energy content of 4700 kcal/kg and high protein content of 47%. According to the Center for Climate and Energy Solutions, algae products including food, fuel and chemical sectors could have a combined annual market value of $320 billion in 2030 [25]. 

Various algae species are also capable of CO_2_ sequestration in thermophilic environments. *Chlorella sorokiniana* demonstrated photosynthetic efficiency and CO_2_ fixation at temperatures above 45 °C. *Chlorella sorokiniana* also produced polymers that can serve as additive in food and cosmetics. *Chlorella* sp. strains were also reported to remove up to 40% CO_2_ in thermophilic temperature ranges [26]. Huesh et al. [27] isolated microalga from a natural alkaline hot spring at a temperature in excess of 60 °C, which demonstrated the ability of reasonable CO_2_ fixation even at such high temperatures. *Spirulina* sp. is a cyanobacterium, commonly known for the use of its biomass in human diet and animal feed. A CO_2_ reduction rate of up-to 105 mgL^−1^d^−1^and more than 15% of CO_2_ removal efficiency were reported by use of *Spirulina* sp. in a 2 L vertical tubular photobioreactor, with illumination conditions of 41.6 µmolm^−2^s^−1^ incident intensity and 12:12 h cycle photoperiod [28]. Superior performance of this algae strain was recorded in the presence of a chemical adsorbent; thus algae can be used in combination or as part of a process train with other physical and/or chemical CO_2_ sequestration processes. Table 1 lists a summary of various algal species and their respective growth rates, yields, and CO_2_ removal rates at specific CO_2_ concentrations.

## 3. Types of Algae Bioreactors

**Figure 1:**
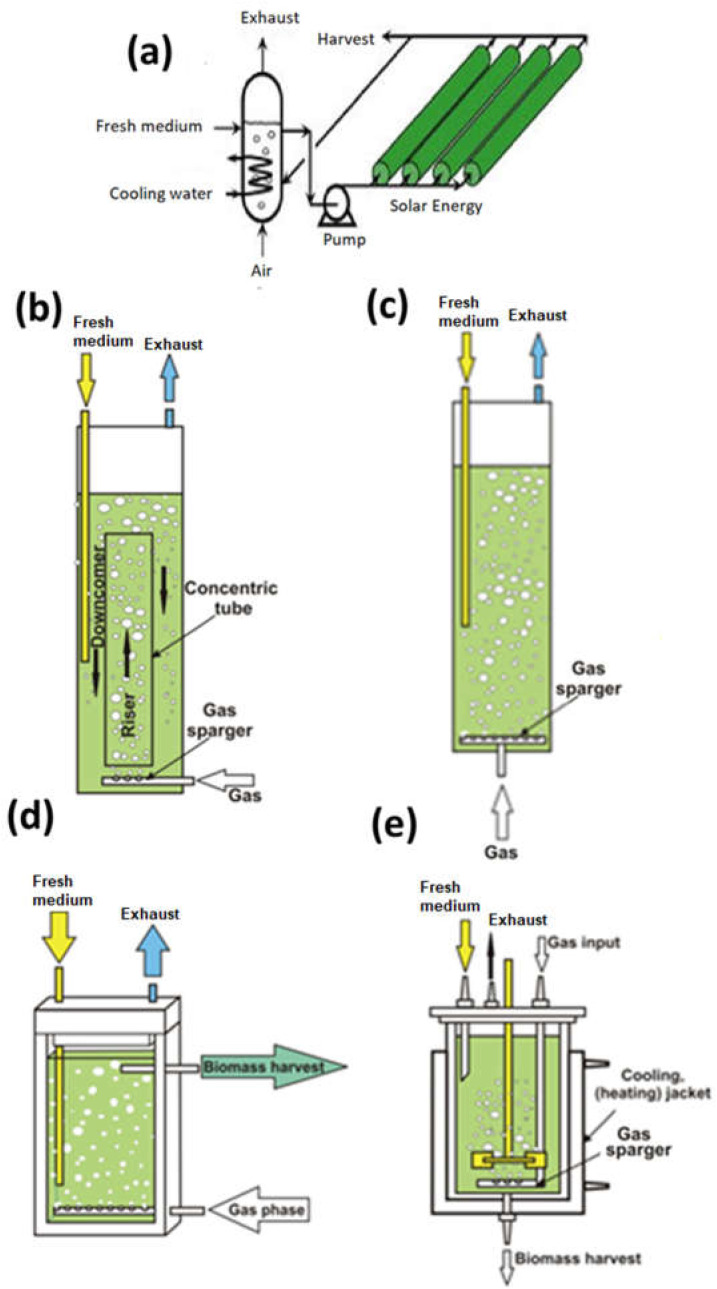
Schematic diagrams of main bioreactors (**a**) Tubular (Growing Algae, [32]), (**b**) Vertical Air-lift (Płaczek et al. [33]), (**c**) Bubble column (Płaczek et al. [33], (**d**) Flat panel (Płaczek et al. [33]), and (**e**) Stirred tank reactor (Płaczek et al. [33]).

Bioreactors are enclosed vessels designed for cultivating biomass in a controlled manner. Cultivating algae in a bioreactor could optimize growth which leads to more efficient CO_2_ removal from the flue gas. There are three main categories of bioreactors used for CO_2_ sequestration: tubular, column, and flat plate. Based on its positioning and mass transfer methods used, they can be further categorized into several types. Column bioreactor can have mixing effect through airlift, bubbling, or mechanical stirring. Figure 1 provides schematic diagrams of main bioreactors discussed below.

### 3.1. Tubular Bioreactor

Tubular bioreactor is the most commonly used bioreactors. They comprise of long, cylindrical tubes that can be arrayed vertically, horizontally and as a helix. Scaling up tubular bioreactor can be done by increasing tube length or diameter of the tubes. However, there is a limit on how much length and diameter can be increased. Longer tubes have higher potential for oxygen accumulation within the reactor. High concentrations of oxygen can inhibit the growth of photosynthetic microorganisms [29]. Larger diameter tube is a better solution if sufficient illumination is available [30]. In addition to oxygen accumulation, fouling and high energy consumptions are the two main concerns with tubular reactors. Fouling of algal cells on the wall of tubular bioreactors reduces light permeability and hinders biomass productivity. To reduce fouling, flow rate of liquid can be increased, but this requires large amount of energy. 

The advantages of vertical tubular bioreactor include high volumetric and biomass density and less land area requirements. However, scalability of this type reactor is difficult and fouling on tube is also a problem. Horizontal tubular bioreactor is ideal for outdoor applications and the inclination can be easily adjusted to capture sunlight; thus, it has good photosynthetic efficiency, however, the energy consumption for this reactor is higher as compared to vertical tube bioreactor. Furthermore, large area requirement and oxygen accumulations are some of the few drawbacks. The advantages of helical tube bioreactor include high photosynthetic efficiency and efficient mass transfer of CO_2_ from gas phase to liquid phase. However, the energy consumption and fouling are high for this type of reactor and scalability is limited. 

### 3.2. Vertical Airlift Reactor

Vertical airlift reactor is a modified bubble column reactor. It consists of vessels that contain a riser, a gas separator, and a down-comer in each vessel. The gas separator and the down-comer are interconnected. The gas is injected from the riser creates a turbulent flow of fluid. The fluid goes up to the separator region where the gas disengages from the fluid. After separation, liquid returns to the bottom of the vessel through the down-comer. Light could be provided internally in the riser section or externally. This provides good light/dark cycles that are essential for photosynthesis and algal growth. Vertical airlift bioreactor has many advantages including good exposure of cells to light, high gas exchange, and ease of scalability. However, compared to tubular reactor, vertical airlift reactor has lower biomass density.

### 3.3. Bubble Column Reactor

Bubble column reactor is simpler than vertical airlift reactor. It usually consists of vertical vessels that contain a sparger at the bottom of each vessel. The sparger delivers mixing and CO_2_ mass transfer without creating mechanical stress to algal cells. In a scaled-up version, bubble column is very tall and perforated plates are used to break up and redistribute coalesced bubble [5]. In bubble column reactor, light is usually provided externally. Bubble column and airlift bioreactors have similar productivity, but at higher gas velocities bubble column outperforms airlift reactor. Bubble column reactor can be scaled up easily and it has low capital and power consumption cost. However, the surface to volume ratio and biomass density are lower for this type reactor as compared to tubular reactors. 

### 3.4. Flat Panel or Plate Reactor

Flat panel or also known as flat plate reactors have cuboidal shape and can be arranged vertically or horizontally. They have gained interest because of their high surface to volume ratio which provides excellent illumination for photosynthesis. Horizontal flat plate receives more light but takes up space. Vertical flat plate is more effective with respect to land usage but requires more rigid and expensive materials to build [31]. The width of flat plate reactor is usually thin (1.5–3 cm) so as to provide short light path and improves productivity. Flat panel reactor has many advantages including high photosynthetic efficiency, low oxygen accumulation and low power consumption. The drawbacks of this reactor include short light path which limits culture volume and high aeration rate which creates shear stress on algal cells.

### 3.5. Stirred Tank Reactor

Stirred tank reactor is a conventional reactor where agitation is provided mechanically by impeller. Flue gas containing large amount of CO_2_ is supplied from the bottom of the reactor as a carbon source [34]. An example of such reactor is Fermenter type algal photobioreactor. These are mechanically stirred vessels in which algal strains are cultivated with artificial or natural lighting. Such systems are usually capable of offering only low biomass productivity and CO_2_ fixation [33]. Stirred tank reactor can be operated in batch, semi-batch or continuous mode. In batch stirred-tank reactor, reactants are fed, and products remained in the tank until for a certain time period. In continuous stirred-tank reactor, there are inflow and outflow of reactants and products simultaneously. Stirred tank reactors have uniform mixing and good temperature control, however vigorous stirring could damage sensitive cells. Furthermore, surface area to volume ratio is lower for stirred tank reactor. 

Bioreactor applications for CO_2_ removal should consider productivity, power consumption, and scalability. Productivity is affected mainly by light, mixing of carbon source and removal of oxygen accumulation. High productivity ensures more effective CO_2_ removal. It is important that losses (i.e., “off-gas” loss of CO_2_) must be accounted for in removal efficiency calculations when experimental data are compared. Power consumption determines if the chosen bioreactor could be a net CO_2_ absorber. Scalability allows the reactor to have a more industry-wide impact than just a laboratory scale. Considering the above factors, vertical airlift, bubble column and flat panel bioreactors have the potential to be used in large-scale applications for CO_2_ removal. 

### 3.6. Other Types of Bioreactors

The literature also reports other designs for algal bioreactors that do not exactly fall within the spectrum of the algal bioreactor classification as mentioned above. As in the case of various environmental remediation and purification applications, membrane technology also found its use in algal photobioreactors. Kumar and co-workers [35] utilized a hollow fibre membrane photobioreactor for removal of CO_2_ from a gas mixture and reported a CO_2_ removal efficiency of 85% with a fixation rate of 60 g/m^3^h. Fixation refers to the process by which inorganic carbon (i.e., CO_2_) is converted to organic compounds by plants and autotrophic organisms. Fan et. al. [36] report that membrane algal reactor showed a CO_2_ fixation rate up to 0.95–5.4 times greater than airlift and bubble type photobioreactors utilizing *Chlorella vulgaris* strain. The membrane photobioreactors can also vary in terms of the membrane shape and features. Zhang et. al. [37] report use of algal-bacterial consortium on a flat sheet of polyvinylidene fluoride (PVDF) membrane in a 10.35 L bioreactor to remove organic carbon content from wastewater.

**Table 2:** Summary of different types of bioreactor for testing Algal species with respect to biomass yield and CO_2_ fixation efficiency and removal rates.

Type of Bioreactor	Algal Species	Invention Year & Country	Biomass Yield ^a^ (%)	CO_2_ Fixation Efficiency (%)	CO_2_ Removal Rates (gCO_2_ L^−1^ day^−1^)	References
Tubular	*Spirulina* sp. ^b^	2016 & Brazil	550		0.197 ± 0.061	da Rosa et al. [28]
Bubble column	*Parachlorella kessleri*	2022 & Canada		0.49%	0.211	Beigbeder et al. [20]
Vertical Column	*Chlorophyta*	2021 & Philippines	43.3 ^c^ 31.3 ^d^	15.6	3.91–22.04 3.55–15.66	Alarde et al. [22]
Flat plate	*Chlorella vulgaris*	2011 & China	42 ^e^			Feng et al. [38]
Hollow fiber membrane	*Spirulina platensis*	2009 & USA	63.9	80	1.44	Kumar et al. [35]
Airlift Bioreactor	*Chlorella vulgaris*	2020 & India			0.32 ± 0.01	Madhubalaji et al. [11]

^a^ (C_f_ -C_i_)/C_i_, where C_f_ and C_i_ are the initial and final biomass concentrations; ^b^ With 0.41 mmol L^−1^ Mono ethanol amine (MEA); ^c^ Trial 1; ^d^ Trial 2; ^e^ Corresponding lipid content of *Chlorella vulgaris.*

Sebestyén et al. [39] report a type of reactor in which microalgae are cultivated on disks, partially submerged in water or growth media, rotating on a horizontal shaft. As compared to conventional photobioreactors, this design offers several advantages including a low water requirement, convenience of harvesting, and low footprint. They are also energy efficient, do not require continuous aeration and offer high CO_2_ capture efficiency [40]. 

Microbial fuel cells have long been established as a popular study subject in the scientific community due to their simultaneous environmental remediation and bioelectricity generation potential. A variant of microbial fuel cell typically known as photosynthetic algae microbial fuel cell (PAMFC) has been proposed in several studies for simultaneous treatment and power generation. Li et al. [41] report microalgae strains (*Chlorella vulgaris*) were used for removal CO_2_ in a bubbling-photosynthetic algae microbial fuel cell (B-PAMFC) with CO_2_ fixation of up to 2.8 g/L. In addition, an airlift photosynthetic algae microbial fuel cell (A-PMFC) yielded a CO_2_ fixation rate up to 1149 mg L^−1^d^−1^. Other algal photobioreactors can also be found in the literature such as pyramid type algal photobioreactor, which is a variant of flat panel and airlift systems and is anticipated to offer several times greater productivity as compared to their conventional counterparts [33]. 

Placzek et. al. [33] report a hybrid system consists of closed and open algal reactors in a process train. Algal strains are first cultivated in the closed reactor and then moved to an open system. This method avoids the risk of contamination in initial growth stage and facilitates higher growth rate associated with open systems in the second stage. Xu et. al. [42] report an application of a photobioreactor bag (PBRB) for removal of chemical oxygen demand (COD) in addition to the removal of CO_2_ from biogas produced from piggery wastewater. Table 2 provides a summary of different types of bioreactors for testing algal species with respect to biomass yield and CO_2_ fixation efficiency and removal rates.

## 4. Factors Controlling CO_2_ Removal: Focusing on Light Intensity

In this section, the effects of light intensity, light incident, photoinhibition, light supply arrangements and photoperiod on the efficiency of CO_2_ removal in algae bioreactors are discussed. Light incident is the light that falls on an object and it can be from natural lighting, or from a light source. The light intensity is the energy that is transmitted per unit area per time. The incident angle refers to the angle between the direction of the light and the surface. Table 3 provides a summary of the influence of light intensity and photoperiods on the biomass productivity of various algal species in a variety of bioreactors.

### 4.1. Effect of Incident Light Intensity

CO_2_ removal and subsequent algal growth is a complicated process, which is also influenced by the incident light among host of other parameters. There is a lack of consensus on the exact relationship between light intensity and the CO_2_ fixation, but it has been observed that the increase in light intensity also cause an increase in CO_2_ fixation, until the light saturation is achieved. When the gaseous stream containing CO_2_ is bubbled through the algal media in an algal PBR, the CO_2_ present inside the gas bubbles must be reasonably dissolved into the microalgae suspension to undergo the light driven photosynthetic conversion into complex organics. Hence, the influence of incident light on each micro-algal cell is a function of internal mixing. Sufficient mixing is required to assist the CO_2_ in bubbles to cross over the gas liquid interface, as well as provision of equal light to all algal cells. The attenuation of incident light is also a concern as absorption and scattering of light take place along the path, mainly due to micro-algal cells themselves. Self-shading effects have also been observed in algal PBRs, especially when the system is illuminated using sunlight. Microalgae are capable of employing 50% of the sunlight for effective conversion to complex organics via photosynthesis, with rest of the sunlight being wasted. While it is possible to significantly improve microalgae growth by utilizing artificial light sources (usually employed in case of a closed type algal photobioreactors), the associated costs might make this option rather unattractive as compared to natural sunlight. LED light sources are usually preferred over fluorescent lamps due to longer lifespans and lesser energy footprint [43].

**Table 3:** Influence of light intensity and photoperiods on the biomass productivity of various Algal species in a variety of bioreactors.

Type of Bioreactor	Algal Species	Light Source	Light Intensity (µmol m^−2^s^−1^)	Photoperiods	Biomass Productivity (mg L^−1^ day^−1^)	References
Photobioreactor (Volume: 10 L, Diameter: 22 cm, Height: 29 cm)	*Arthospira (Spiralina) plantesis*	White LED lamp	635, 980, 1300, 2300	Light-dark cycles (12 h:12 h)	620	Chaiklahan et al. [13]
Flat plat (Volume: 4 L, Length: 33 cm Diameter: 22 cm, Height: 10 cm)	*Oscillatoria* sp.	Fluorescent lamps	160 µE m^−2^s^−1^	Light-dark cycles (12 h:12 h)	75 (Once supply frequency) 80 (Twice supply frequency) 92 (Thrice supply frequency)	Nithiya et al. [22]
Photobioreactor (Volume: 30 L, Diameter: 40 cm, Height: 50 cm)	Mixed culture	LED lights (red, blue, and white)	110	Light-dark cycles (16 h:8 h)	47.75 (UQ-Lake A at 10% CO_2_) 50.04 (UQ-Lake F at 10% CO_2_) 54.87 (Strom water A at 10% CO_2_) 54.5 (Strom water F at 10% CO_2_)	Aslam et al. [44]
Sintered disk glass bubble column	*S. Platensis*	White LED light		Light-dark cycles (14 h:10 h)	457.5 (Maximum with CO_2_ and NaOH for culture medium of Z(20)3)	Kumari et al. [45]
In-vertical tubular photobioreactor (Volume: 2.5 L)	*Chlorella vulgaris (BA 002)*	High pressure sodium (HPS) light LED lights (red, blue, and white)	13.5 µmol s^−1^	Light-dark cycles (12 h:12 h, 18 h:6 h, 24 h:0 h)	27.08 ± 7.80 (under optimum HPS) 24.21 ± 8.89 (under optimum LED)	Ratomski and Hawrot-Paw, [46]
	*Scenedesmus Abundans*	White tube light	27 (BG-11 media) 40.5 (Fogg’s media) 54 (CHU-13 media)	Light-dark cycles (16 h:8 h)	47.4 ± 0.002 (BG-11 media) 70.23 ± 0.001 (Fogg’s media) 52.46 ± 0.002 (CHU-13 media)	Rai and Gupta, [47]
Photobioreactor (Volume: 40 L)	*Chlorella vulgaris*	LED light	0–80			Geiman et al. [48]
Sequential Column (Working volume: 300 mL Diameter: 56 mm Height: 160 mm)	*Chlorella PY-ZU1*	White lights and plant lights	4500–6000 lux		950 (at 10 min of Empty Bed Residence Time)	Cheng et al. [49]

### 4.2. Photoinhibition and Incident Light Intensity

The duration of exposure, as well as the intensity of incident light also influence the performance of algal bioreactor. Algal PBR is often divided into three major regions, which are photo-inhibition, photo-limited, and stagnated region. The region where the light intensity is more than the saturation limit leads to the decline in photochemical efficiency of microalgae. This region is termed as photo-inhibition region. Chaiklahan et al. [13] report that the highest light intensity at which reasonable algal biomass growth was observed is around 2400 µmol/m^2^s. While in most parametric studies done, light intensity never exceeded 1000 µmol/m^2^s. Tyystjärvi [50] reports that photoinhibition can be irreversible, which is ‘light induced loss of oxygen evolution and electron-transport ability’ of the chlorophyll [50]. When the light intensity is reduced below the saturation level, the photosynthetic efficiency can be recovered [51]. This second type of reversible photoinhibition is also termed as ‘dynamic’ photoinhibition [50]. The potential reason behind the irreversible photoinhibition is the damage to type II photosystem. Type I and type II photosystems are algae/plant-based protein complexes that comprise of various types of chlorophyll. Type I photosystems are capable of absorbing longer wavelengths of incident light, while type II photosystems absorb the shorter one, generally below 690 nm wavelength, and are more sensitive to incident light. Type II photosystems get continuously damaged and repaired during the normal course of life of photosynthetic organisms. This damage is typically higher in magnitude when the incident light intensity exceeds the saturation level. However, the damage to type II photosystems does not occur when the light intensity is too high and can also take place when the photosynthetic organisms is exposed to low intensity light continuously [52]. The type I photosystems are not immune to light induced damage. However, the effects of induced light are rather slow in type I photosystems. It is worth mentioning that even the ‘irreversible’ photoinhibition is not always permanent due to inherent repair mechanisms. Forster et al. [53] report a reason behind the photoinhibition is the increase in oxidative stress due to excess light intensity. Excess light intensity can cause an augmented generation of reactive oxidation species namely radical species with very high oxidation potential. 

### 4.3. Effect of Light Provision Scheme

Another way to improve the provision of incident light lies in the design of the algal PBRs. Designs with higher surface to volume ratio such as tubular algal PBR and flat plate PBR have been fabricated using materials with high transparency materials to improve the incident light. Another novelty in the design that address this issue is the liquid foam bed PBRs, which employ foam to expose micro-algal cells to light. As compared to plants, algae need less intensity of light for their growth [43]. In high temperature conditions, lower intensity of light have been reported as suitable for algal growth, as compared to colder conditions. Excess production of chlorophyll has also been reported to decrease the penetration depth of incident light. Cheng et al. [49] report that by optimizing the light intensity, algal growth can be doubled. At 6000 lux, peak growth rate performance was achieved faster as compared to 4500 lux. 

### 4.4. Effect of Photoperiod 

Another important parameter to be considered is the duration and pattern of exposure (i.e., period of time each day during which an organism receives illumination), usually termed as photoperiod. Among other factors, different photoperiods have also been tested to design the optimum strategy of light exposure, including 12/12 [22,54], 16/8 [44] and 14/10 [45] hour light/dark cycles. Influence of algal growth in a photobioreactor is also a function of type of light source used [46]. It is worth mentioning that continuous exposure to light (24/0 light/dark cycle) has led to inferior performance of the algal photobioreactor; thus dark period is also required by the algal species for formation of ATP and NADPH [47]. The energy consumption due to continuous provision of light during the light period can be brought down significantly by design and deployment of control system that affects the incident light intensity as per the CO_2_ concentration using a feedback control loop [48]. 

## 5. Conclusions

The CO_2_ sequestration by algal photobioreactor and the parameters influencing the process are frequently studied in peer reviewed literature. There are still gaps for more research in certain areas. In this work, we reviewed recent developments of algal bioreactors used for CO_2_ removal and the factors affecting the reactor performance. The main focus of the study is related to intensity of light and photoperiods. The influence of light intensity has been widely studied in case of bubbling and airlift algal photobioreactors, however future research should focus on the influence of light intensity and optimum photoperiod in novel designs of algal photobioreactors such as algal microbial fuel cell, algal disk type reactors and novel membrane type algal bioreactors. While algal photobioreactors are a cleaner alternative of CO_2_ fixation as compared to their chemical counterparts, there is a dire need for development of schemes to deploy algal photobioreactors more frequently in industrial CO_2_ sequestration processes. In future, novel hybrid processes in which micro-algal strains with commercial value will be developed in addition to CO_2_ removal. An interesting application would be removal of CO_2_ from a space vessel’s atmosphere and provide oxygen and edible biomass to the personnel onboard [55]. The improvement in photosynthesis efficiency will also allow the development of practically viable portable and/or modular algal bioreactors. The practical design of modular algal photobioreactors cannot be achieved without the improvement in the utilization of incident light intensity. While there are efforts underway to optimize the light sources and the incident light frequencies, further investigations are required that might have good potential of rapid CO_2_ fixation. Since utilization and commercial use of algae is fast growing and has a broader significance on society, a more research on novel algae bioreactor development and factors that influence algae growth in such reactors are expected.

## Figures and Tables

**Table 1:** Summary of various Algal species and their respective growth rates, yields, and CO_2_ removal rates at specific CO_2_ concentrations.

Algal Species	Growth Rate (mg L^−1^ day^−1^)	Biomass Concentration (g L^−1^)	CO_2_ Concentration *v*/*v* (%)	CO_2_ Removal Rate (gCO_2_ L^−1^ day^−1^)	CO_2_ Removal Efficiency (%)	References
*Botryococcus brauna 765*		2.31 on 25th day	20			Ge et al. [7]
*Chlorella vulgaris*	242		9			Ghayal [9]
*Chlorella vulgaris*	171	1.13 ± 0.03	15	0.32 ± 0.01		Madhubalaji et al. [11]
*Scenedesmus obliquus*	4.36 g m^−2^day^−1^		10		40	Sun et al. [12]
*Arthospira (Spiralina) plantesis*	620					Chaiklahan et al. [13]
*Chlorella vulgaris*		2.12 (BCR) ^a^ 1.42 (ALR) ^b^			40 25	Alhaboubi et al. [14]
*Chlorella sorokiniana TH01*	284–469		5		63–100	Do et al. [15]
*Chlorophyta*	0.56 g in^−2^ day^−1,c^ 0.51 g in^−2^ day^−1,d^	21.5 g in 7 days ^c^ 19.7 g in 7 days ^d^			22.04	Alrade et al. [16]
*Chlorella vulgaris*		1.01	15		80	Senatore et al. [17]
*Chlorella* sp.	6.7 g m^−2^day^−1,e^ 28.0 g m^−2^day^−1,f^					Feng et al. [18]
*Chlorella vulgaris*	7 ± 1	0.572 ± 0.04		0.927 ± 0.073		Ratomski et al. [19]
*Parachlorella kessleri*	104		5	0.211		Beigbeder et al. [20]
*Chlorella sorokinaina*	0.292 ± 1 ^g^ 313 ± 5 ^h^					Cui et al. [21]
*Chlaymydomonas reinhardtii*	369 ± 10 ^g^ 329 ± 12 ^h^					Cui et al. [21]
Mixture of *Chlorella* sp., *Scenedesmus* sp., and *Ankistrodesmus* sp.				0.979	59.8	Rinanti et al. [23]
*Spirulina* sp. ^i^	110.2 ± 4.2	1.30 ± 0.07		0.197 ± 0.061	29.8 ± 0.9	Da Rosa et al. [28]

^a^ BCR is Belt Conveyer Reactor; ^b^ ALR is Air Lift Reactor; ^c^ Trial 1; ^d^ Trial 2; ^e^ Indoor membrane bioreactor. ^f^ Outdoor membrane bioreactor; ^g^ Original sparger design; ^h^ Modified sparger design; ^i^ ith 0.41 mmol L^−1^ Mono ethanol amine (MEA).
